# 

**Published:** 1887-10

**Authors:** 


					﻿Ensdory sadre dagsr akdye 
Vol. III
OCTOBER 1887
No. 4
DANIEL'S
MEDICAL
JOURNAL
L. M D ahste hagst aswi dehi
Noasr hhstweu gafserw 
Sarrwei gafsr daswer agstwl gafsre
				

